# Hi-fidelity discrimination of isomiRs using G-quadruplex gatekeepers

**DOI:** 10.1371/journal.pone.0188163

**Published:** 2017-11-16

**Authors:** Nianjia Seow, Renzo A. Fenati, Ashley R. Connolly, Amanda V. Ellis

**Affiliations:** 1 Flinders Centre for Nanoscale Science and Technology, Flinders University, Bedford Park, SA, Australia; 2 School of Chemical and Biomedical Engineering, The University of Melbourne, Parkville, Victoria, Australia; Northeastern University, UNITED STATES

## Abstract

Core microRNA (miRNA) sequences exist as populations of variants called isomiRs made up of different lengths and nucleotide compositions. In particular, the short sequences of miRNA make single-base isomiR mismatches very difficult to be discriminated. Non-specific hybridizations often arise when DNA probe-miRNA target hybridization is the primary, or initial, mode of detection. These errors then become exacerbated through subsequent amplification steps. Here, we present the design of DNA probes modified with poly-guanine (PG) tracts that were induced to form G-quadruplexes (G4) for hi-fidelity discrimination of miRNA core target sequence from single-base mismatched isomiRs. We demonstrate that, when compared to unmodified probes, this G4 'gate-keeping' function within the G4-modified probes enables more stringent hybridization of complementary core miRNA target transcripts while limiting non-specific hybridizations. This increased discriminatory power of the G4-modified probes over unmodified probes is maintained even after further reverse transcriptase extension of probe-target hybrids. Enzymatic extension also enhanced the clarity and sensitivity of readouts and allows different isomiRs to be distinguished from one another via the relative positions of the mismatches.

## Introduction

IsomiRs are closely related variations of a miRNA, made up of nucleotide substitutions, additions or deletions to core miRNA sequences [1,2]. Increasingly, isomiRs are found to have physiological and evolutionary importance, playing very specific roles in cellular regulation, many of which are still being discovered [3–5]. Thus, there is interest in studying different aspects of isomiRs, such as their up-/down-regulation or interactions with other biomolecules, and this necessitates the accurate and efficient detection of different, specific isomiRs [6,7]. Due to low miRNA copy numbers, analysis of miRNA is often focused on improving the sensitivity of detection and as a result modes of miRNA amplification such as enzyme cascades, real-time polymerase chain reaction (RT-PCR), loop-mediated isothermal amplification and rolling cycle amplification [8–10] have been employed. These detection methods almost always involve an initial hybridization step by probes/primers. The detection of isomiRs is further complicated by their length (20–24 nt) and the inability to accurately detect single nucleotide variations through probe/primer hybridization, which is often prone to errors such as non-specific binding between closely-related sequences [11,12]. Subsequent polymerase-mediated amplification steps then exacerbate these erroneous hybridizations, bringing about false positive detections. However, several techniques have been designed to improve isomiR detection specificity such as optimizing the probe-target ratio [13], the use of T4 ligase, and also multiple primers [10,14–16]. These increase the complexities of any design (that is, the need for multiple primers and enzymes) and necessitate multi-step detection processes. In addition, they remain challenged by the need to discriminate with hi-fidelity isomiRs with single-base variations.

G-quadruplexes (G4) are non-canonical DNA structures formed when tracts of guanine bases are arranged first in tetrads, held by Hoogsteen hydrogen bonds, and then stacked into quadruplexes [17,18]. Similar to miRNA, G4 also plays a key role in cellular regulation. One regulatory, or gate-keeping function, of G4 involves its formation at the ends of telomeres and promoter sites, which prevent uncontrolled cellular proliferation or oncogenic activations [19–21]. G4 has inspired various diagnostic platforms that have leveraged the (dis)assembly of reporter-tagged G4 for the screening of cations (e.g., Na^+^, K^+^), and ligands for therapeutic applications such as telomere-targeting cancer drugs [22,23]. G-quadruplexes also have enhanced stability in comparison to hydrogen-bonded DNA base-pairs, exhibiting higher melting temperatures (T_m_’s) (between 63°C to 61°C favoring intramolecular G4, at comparable conditions), and generally formed more readily than DNA duplexes, especially at conditions of higher temperatures, lower pH and in the presence of stabilizing ligands and monovalent ions such as K^+^ ions [24]. Stability of G4 is also dependent on the nature of the folding (intramolecular G4 forms from a single strand with multiple stretches of guanine repeats folded upon itself while intermolecular G4 conformation is made up of two or more strands), orientation of the strands (parallel and anti-parallel), and presence of ligands and K^+^ ions [25].

DNA modified with G4 and fluorescent reporters have also been incorporated into miRNA detection platforms, whereby they have been shown to mediate DNA probe-miRNA target binding and transduction of readouts. For example, Zhou and co-workers have shown the use of a G4-molecular beacon (MB) reporter successfully reduced miRNA binding mismatches from 24% to 6% while also bringing about improved signal amplification [26]. In our previous work we developed a G4-MB platform, wherein the detection of target miRNA resulted in a dual response of a G4-mediated gold nanoparticle assembly and MB fluorescence restoration, which could more accurately represent the miRNA binding event [27].

The design of molecular probes that bring about sensitive and selective detection remains a foremost consideration when detecting targets with short sequence lengths and low abundances such as miRNA. Rigorous detection of these sequences is further complicated by the presence of isomiRs.

In this work, we mitigate the complexities of detection strategies such as using multiple probes or extensive amplification steps and focus on the inclusion of poly-guanine (PG) tracts into the design of the DNA molecular probes for isomiR target detection. These PG tracts then form G4 gate-keepers allowing for more stringent target detection. By designing and using G4 as gate-keepers the effect of the DNA probe-miRNA hybridization equilibria was monitored for more favorable discrimination of core miRNA targets against its isomiRs, in comparison with unmodified, base-pairing probes commonly used.

The premise of gate-keepers was further challenged with subsequent extension of the probe-target hybrids using a reverse transcriptase, whose action also enhanced the clarity of the readout. The enzymatic extension also allowed different isomiRs to be distinguished from one another based on the relative distance of each mismatch from the 3' end.

## Materials and methods

### Materials

Tris(hydroxymethyl)aminomethane (Tris), boric acid, ethylenediaminetetraacetic acid, magnesium chloride and potassium chloride were purchased from Sigma-Aldrich, Australia, and made up to the desired concentrations in Milli-Q water (18 MΩ.cm). The DNA probes used were switching nucleic acid probes [28], guanine-rich (PG tract) SNAP (G-SNAP), G-SNAP with PG tract displaced by one base towards the 3' end (G-SNAP-Alt) and poly-thymine (Poly-T)). The target oligonucleotide miRNAs were *Cel-miR-54* core miRNA, *IsomiR-1*, *IsomiR-2* and *IsomiR-3* (see Table 1 for sequences).

**Table 1:** Sequence of probes (SNAPs) and miRNA targets (*Cel-miR-54* core and its isomiRs). The underlined sequence represents the recognition site (probe for miRNA target). The G-SNAPs contain a tract with five G-bases that brings about intermolecular G4 formation. The bases in bold represent A to U variations in the isomiRs. Probes and targets are 50 and 22 bases long, respectively.

Name	Sequence
*Cel-miR-54* core target	5' AGGAUAUGAGACGACGAGAACA
*Cel-miR-54 IsomiR-1*	5' AGGAUAUGAGACGACGAGAUCA
*Cel-miR-54* *IsomiR-2*	5' AGGAUAUGAGACGUCGAGAACA
*Cel-miR-54* *IsomiR-3*	5' AGGAUAUGUGACGACGAGAACA
SNAP	5' ATCCACGGGCACTGCGAGAGTCAGGATATGCTCTTGT TTCTCGTCGTCTC
G-SNAP	5' ATCCACGGGCACTGCGAGAGTCAGGATATGC**GGGGG**T TTCTCGTCGTCTC
G-SNAP-Alt	5' ATCCACGGGCACTGCGAGAGTCAGGATATGCTC**GGGG** **G**TTCTCGTCGTCT
Poly-T	5' GTACGACTGCGCGGCGTTTTTTTTTTTTTTTTTTTTTTTTTT TTTTTTTTTTTTTT

Both the DNA probes and miRNAs were purchased from IDT DNA Technologies, and reconstituted in 50 mM Tris to 100 μM. Agarose, 3,3',5,5'-tetramethylbenzidine (TMB), SYBR-Safe dye, 10 mM mixed dinucleotide triphosphates (dNTPs) and Moloney murine leukemia virus reverse transcriptase (M-MLV RT) and M-MLV RT buffer were all purchased from Promega, Australia and used as received. Hemin (Promega, Australia) was diluted in DMSO to a concentration of 0.26 μg/μL.

### Hemin-TMB testing of G4 formation

All oligonucleotides (1 μL, 10 μM) were annealed and stabilized in aqueous K^+^ ion solutions (500 mM) in a total volume of 38 μL for 2 h. Thereafter, each sample was mixed with TMB (360 μL), followed by the addition of hemin (2 μL, 0.26 μg/μL). The absorbance intensity was monitored over time on a UV-spectrophotometer (Cary) at 630 nm. All experiments were performed in triplicate.

### Hybridization of DNA probe-miRNA target

Equimolar amounts (1.25 μL, 100 μM basis) of each DNA probe (SNAP (50 b ssDNA), G-SNAP ((50 b ssDNA), G-SNAP-ALT (50 b ssDNA) and Poly-T) was mixed with each miRNA oligonucleotide target (*Cel-miR-54 core* miRNA, *IsomiR-1*, *IsomiR-2* and *IsomiR-3*) in 50 mM Tris, 2 mM MgCl_2_ and KCl (addition from a 1 M stock adjusted to the desired concentrations—100, 300 and 500 mM) in a total volume of 25 μL. Each sample was then annealed at 95°C for 2 min and then left to cool to room temperature for 2 h, after which time they were subjected to agarose gel electrophoresis characterization.

### Agarose gel electrophoresis of DNA probe-miRNA target hybrids

5% agarose was prepared in SYBR Safe-stained 0.5x Tris/Borate/EDTA (TBE). The DNA probe-miRNA target hybrids were run at 90 V for up to 2 h, with images acquired using the Image-Lab software (Bio-Rad) at 15 min intervals up to 2 h.

### Determination of DNA probe-miRNA target discriminatory power

The discriminatory power (DP) of the *Cel-miR-54* core target against its isomiRs was determined from the stained gel electropherograms the intensities. Where the intensities of the unbound DNA probe, DNA probe-miRNA target hybrid and G4 bands were quantified using the Image-Lab gel analysis software and the Image J (National Institute of Health) program. The intensity of the DNA probe-miRNA target hybrid band was then expressed as a percentage of the total band intensities within the same lane, given as:

$$\mathrm{Percentage}\;\mathrm{intensity}=\frac{Absolute\;Intensity\;(hybrid\;band)}{Total\;Intensities\;of\;all\;bands\;within\;same\;lane}\times \;100$$

DP is defined and calculated as follow:

$$\begin{array}{l} DP\;of\;Cel-miR-54\;over\;mismatched\;isomiR\\ =\;\frac{Percentage\;Intensity\;\left(Cel-miR-54\;hybrid\;band\right)-\;Percentage\;Intensity\;(isomiR\;hybrid\;band)}{Percentage\;Intensity\;(Cel-miR-54\;hybrid\;band)} \end{array}$$

In the absence of non-specific hybridizations, isomiR hybrids are not expected to be formed or observed. As such, based on the equations above, this gives a DP of 1.0, and this also represents perfect discrimination against mismatched isomiRs (hybrid bands observed only for perfect matched miRNA core targets, but not for mismatched isomiRs). The presence of isomiR hybrid bands decreases the DP from 1.0, until a lower limit of 0.0, which also signifies a complete lack of discrimination of isomiRs (both perfectly-matched miRNA core target and its mismatched isomiRs gave rise to hybrid bands readouts of comparable intensities).

The calculated DP for the G4 modified probes was compared against that of modified probes using a 2-tailed Student's t-test (n 3), and the p-value was determined. This will illustrate how significantly the incorporation of the G4 in the probes affects fidelity of probe-target hybridization.

### Extension by M-MLV RT

G-SNAP and G-SNAP-ALT DNA probes were first incubated with an equimolar amount of miRNA target (*Cel-miR-54 core* miRNA, *IsomiR-1*, *IsomiR-2* and *IsomiR-3*). After 2 h, the DNA probe-miRNA target hybrids (10 μL) were then mixed with dNTPs (1 μL, 10 mM), M-MLV RT buffer (2 μL) and M-MLV RT (1 μL) made up with Milli-Q water to a total volume of 20 μL. The reaction was incubated at 40°C for 1 h and immediately characterized on a SYBR Safe-stained 0.5x TBE 5% agarose gel.

## Results and discussions

### Working principle of the DNA probe-miRNA detection system

Four different DNA molecular probes and four miRNA targets were interrogated in this work (Table 1). The DNA molecular probes were SNAP, G-SNAP, G-SNAP-Alt and poly-T. SNAP is a DNA molecular probe modelled after a dual switching DNA module that our group has previously designed to detect isomiRs with 5' fidelity and that employ RNA reverse transcriptase and DNA polymerase for enhanced detection [28]. Thus, the primary miRNA detection mode for SNAP is through a 13-base recognition sequence at the 3' end of the miRNA target. Here, we add to this SNAP design by modifying the DNA sequence to include a PG tract for G4 formation incorporated at the 5' end upstream of the miRNA-recognition sequence, so called G-SNAP (Table 1). A PG tract of a set of 5 guanines was chosen as this has been previously shown to prevent intramolecular binding and favor intermolecular binding for G4 formation [29]. The PG tract position was then translocated 1-base towards the 3' end relative to the G-SNAP probe to produce the so-called G-SNAP-Alt (Table 1). G-SNAP-Alt allowed for the formation of G4 at a different location along the DNA probe sequence. This also resulted in a 1-base overlap with the miRNA-target sequence, enabling G4 to directly interfere with the miRNA hybridization. As a negative control a randomized 56 base DNA probe with a poly-T tail was also investigated.

The four miRNA targets were, *Cel-miR-54* and its isomers: *IsomiR-1*, *IsomiR-2* and *IsomiR-3* (Table 1). *Cel-miR-54* and its isomers were chosen as the miRNA model in our study as it is commonly used as an internal control, or spiked-in as an exogenous reference, in various miRNA detection studies, as well as in *in vivo* settings [30]. Given the widespread use of *Cel-miR-54* as a reference, it is relevant as a target of study, and can be a basis on which other miRNAs studies can be based. With a *Cel-miR-54* core, the isomiRs were designed with a single base variation located 3, 9 and 14 bases from the 3' end, respectively. The different positions of the mismatches are expected to disrupt the continuity of base-pairing to varying extents, further challenging the target detection and discrimination of mismatches.

**Fig 1:**
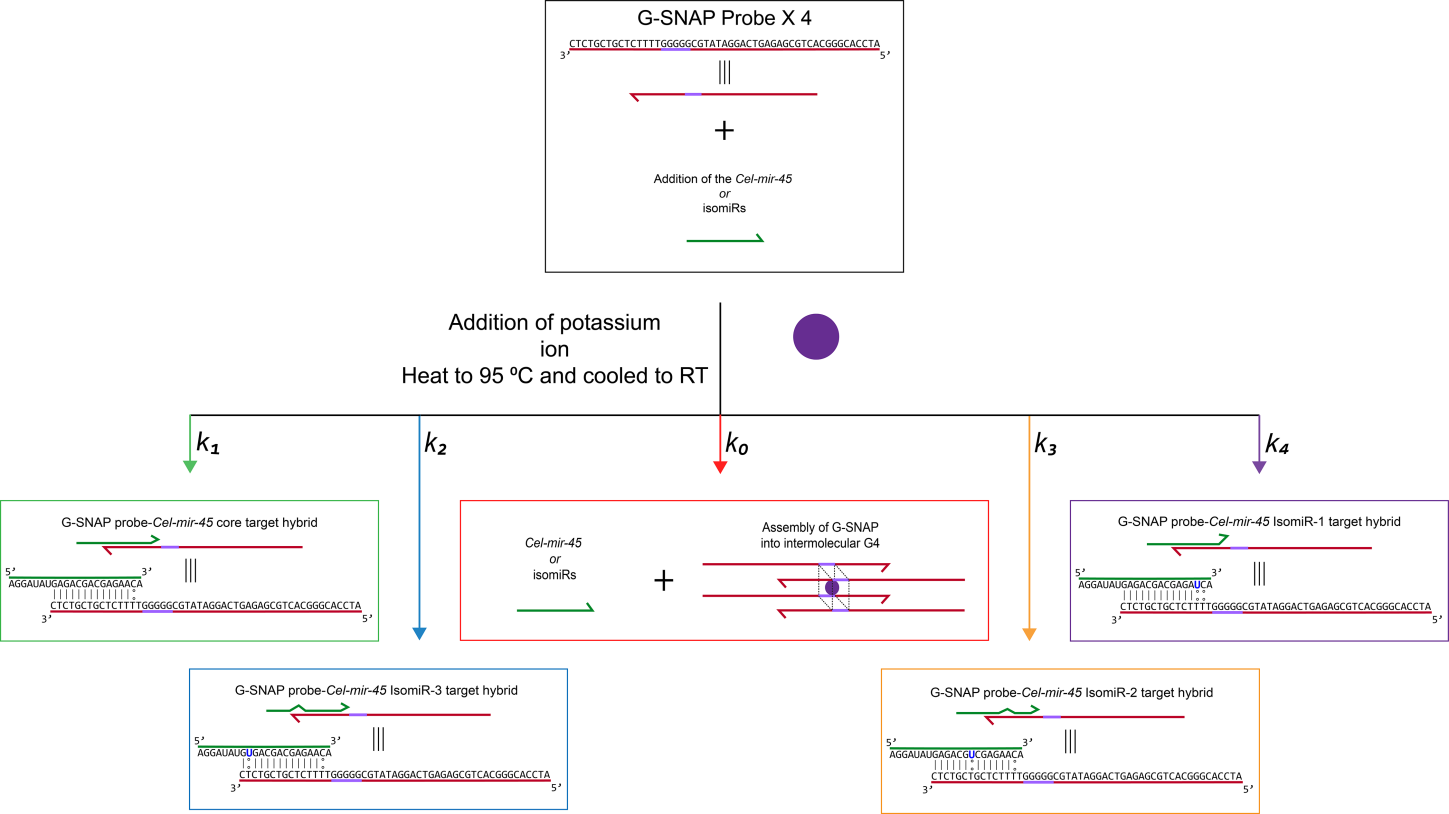
Working principle of G-SNAP in which G-SNAP probes can be hybridized to *Cel-miR-54* core target into stable hybrids (denoted by equilibrium constant *k*_*1*_) or form intermolecular G4 through the association of four G-SNAP strands (*k*_*0*_) in the presence of K^+^ ions. The hybridization of G-SNAP to *IsomiRs-1*, *IsomiR-2*, and *IsomiR-3* are denoted by constants *k*_*2*,_ *k*_*3*,_ and *k*_*4*_ respectively.

In hybridization-based miRNA detections, DNA molecular probe binding to miRNA targets can lead to any one of the following outcomes: (1) specific DNA probe-miRNA target hybrids, (2) non-specific DNA probe-miRNA target hybrids, and (3) remaining as unbound DNA probes. In Fig 1, equilibrium constant *k*_*1*_ represents specific DNA probe-miRNA target (*Cel-miR-54* core) hybrid formation, while *k*_*2*_, *k*_*3*_, and *k*_*4*_ correspondingly represent hybrid formation for *IsomiR-1*, *IsomiR-2* and *IsomiR-3*.

In this work, to address non-specific hybridizations, a PG tract was incorporated into the design of the DNA molecular probes These G-SNAP probes can both hybridize to miRNAs and also assemble into G4 (*k*_*0*_). With the introduction of this competing equilibrium (*k*_*0*_), we sought to exert additional pressure on the G-SNAP-miRNA target hybridization fidelities, such that hybrids which are specific and more stable will be favored (*k*_*1*_), and in turn reduce the extent of non-specific hybridizations (*k*_*2*_, *k*_*3*_, and *k*_*4*_). Essentially, G4 is leveraged to perform a gatekeeping function, and address the lack of fidelity typically associated with miRNA detection techniques that involve direct, base-pairing hybridizations.

The formation of the G4 competes with the miRNA to form the DNA probe-miRNA target hybrid and once formed, has high thermal, and structural, stability due to the Hoogsteen bonds between the four guanines in the G4. This is further stabilized by the stacking of the quartets to a point where G3 is often more stable than G4 because of the possibility of other secondary structures forming i.e., G-wires [31]. For isomiR detection, DNA probe-miRNA target hybrids are known to have higher T_m_’s than DNA:DNA hybrids [32], thus making the determination of a single mismatch much more difficult. By introducing an intermolecular G-quadruplex it is envisaged that the formation of non-specific probe-target hybrids can be further destabilized. This puts strain on the DNA strand as the dimensions of the DNA are stretched to incorporate the quartet. In fact, it has been previously shown that G-quadruplexes can destabilize duplexed DNA, by decreasing the melting temperature of mismatches [33]. In addition, it was found that a 5 base-pair spacing between the G4 site and the base-pairing region is needed to sufficiently mitigate the effect of the former. In our design, due to the close proximity of the PG tract to the miRNA recognition sequence (with ≤ 1 base-pair gap) on the DNA probes, it is highly unlikely that significant DNA-miRNA hybrids and G4 quadruplexes populations will co-exist at the same-time, with the formation of G4 sterically hindering stable DNA-miRNA base-pairing, especially that of mismatched isomiRs.

### Validating the formation of G-SNAP G4 intermolecular adducts

**Fig 2:**
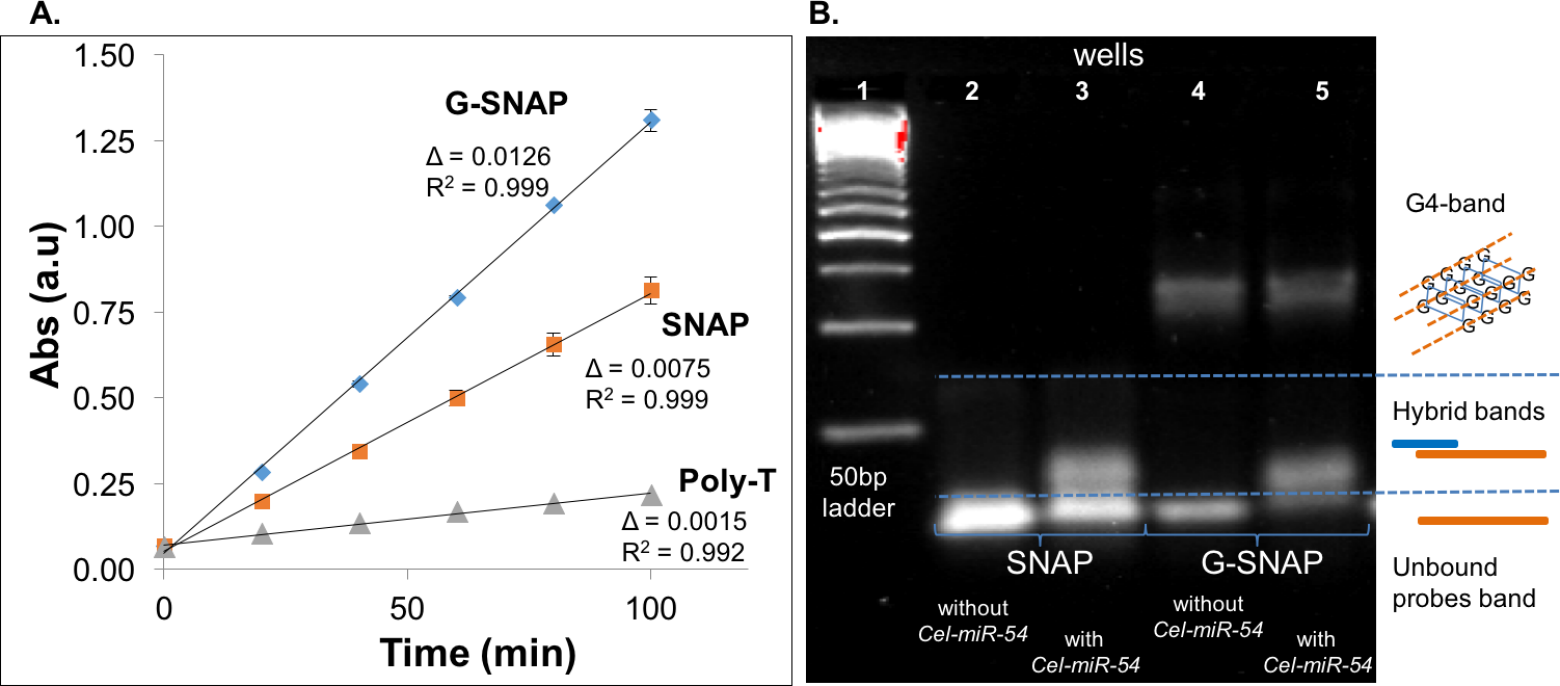
**(A) UV data showing the increase in the absorbance of oxidized TMB substrate in the presence of hemin and G-SNAP, SNAP and poly-T, respectively**; **(B). SYBR-safe pre-stained agarose gel image showing bands and mobility change of SNAP and G-SNAP, with and without *Cel-54* miRNA target, at 100 mM K**^**+**^. Probes and targets were in equimolar ratios.

G4 is known to form complexes with hemin to form G4/hemin DNAzyme with a peroxidase-mimicking activity [32]. Typically, a TMB substrate is used to analyse for this activity through its oxidation to a blue colour (630 nm), detected by a UV-visible spectrophotometer [34]. Three different molecular probes, SNAP, G-SNAP and poly-T (Table 1) were tested on their ability to form DNAzymes. Each was incubated with hemin while monitoring the change in absorbance at 630 nm over 100 min (Fig 2A).

The rate of hemin catalysis is indicated by the slope of each line (Fig 2A). The rate of catalysis exhibited by the G-SNAP probe was 0.0126 a.u. min^-1^, nearly 2 times faster than SNAP (0.0075 a.u. min^-1^) and nearly an order of magnitude faster than poly-T (0.0015 a.u. min^-1^). The increase in absorbance was pronounced for G-SNAP which is capable of forming stable G4/hemin DNAzymes within the 5 guanines in its PG tract. Interestingly, the SNAP also showed an increase in activity with time, albeit moderate in comparison to G-SNAP. This observed increase with SNAP is presumed to be due to the inherent 3 Gs within its sequence (Table 1) which only form marginally stable intermolecular complexes as each probe has only a single tract of short PG (3 guanine) repeats. Conversely, poly-T DNA was unable to form G4/hemin DNAzymes and thus displayed a level of catalytic activity comparable to background oxidation, as expected (Fig 2A).

### Validating the formation of SNAP and G-SNAP DNA probe-*Cel-miR-54* target hybrids

Fig 2B shows a SYBR-safe pre-stained agarose gel electropherogram for the SNAP (50 b ssDNA) and G-SNAP (50 b ssDNA) probes, with and without the *Cel-miR-54* miRNA core target. The probes and targets were in equimolar ratios. The bands presence, intensities and mobilities give an indication of the formation of the DNA probe-miRNA target hybrids, and their relative amounts, as determined through the intensities of the bands. The SNAP probe without the *Cel-miR-54* core target migrated quickly through the gel as a single, defined band (Fig 2B, Lane 2), which we attributed to unbound SNAP. This also reaffirmed the hemin -TMB results (Fig 2A), in that the 3Gs in SNAP did not bring about significant, stable G4 formation, else another slower-moving band will be observed. After the introduction of the *Cel-miR-54* core target a second less mobile adduct appeared, corresponding to a molecular weight similar to that of the expected SNAP-*Cel-miR-54* core target hybrid (Fig 2B, Lane 3). The decrease in the intensity of the unbound probe band (relative to Fig 2B, Lane 2) was also representative of the DNA probe-miRNA target hybrid equilibrium, indicating that probe-target hybrid formation was favoured.

The G-SNAP probe without the *Cel-miR-54* core target is shown to migrate as two distinct DNA adducts (Fig 2B, Lane 4). The faster migration band of the G-SNAP probe has a similar migration rate to that of the unbound SNAP probe, and we associated this with G-SNAP in the unbound conformation. Another band of slower mobility was observed at around 120 bp (Fig 2B, Lanes 4 and 5), which we refer to as the G4 band. As G-SNAP contains a only one PG stretch on a single DNA strand, only intermolecular G4 is possible. The G4 complex will be an assembly of four G-SNAPs (four 50 b sequences) and is likely to exhibit a similar mobility as that of 120 bp dsDNA. Furthermore, the appearance of this G4 band reinforces the proposed *k*_0_ equilibrium (Fig 1) in which G-SNAP probes shift between the unbound and G4 conformations. Once the position of the unbound G-SNAP and G4 was established the G-SNAP probe was added in equimolar amounts to the *Cel-miR-54* core target. Two bands appeared (Fig 2B, Lane 5), but unlike that of the G-SNAP-only (Fig 2B, Lane 4) the faster mobility band now appeared at a position slightly retarded in comparison to the unbound G-SNAP band, and corresponded to the position of the DNA probe-miRNA target hybrid band (Fig 2B, Lane 3). A faint unbound G-SNAP band was observed while the secondary slower G4 intermolecular adduct band was also observed (Fig 2B, Lane 5) at around the 120 bp position. However, the intensity of this G4 band was observed to be lower than its counterpart (G4 band, Lane 4). This can be attributed to the addition of *Cel-miR-54* and introduction of the competing *k*_*1*_ equilibrium (Fig 1) which affected *k*_*0*_ (G4 formation). The continued observation of the G4 band, coupled with the observation of the hybrid band and reduced intensity of the unbound G-SNAP probe band, suggested that the G4 assembly remained stable even after *Cel-miR-54* addition. The competing *k*_*0*_ *and k*_*1*_ equilibria were both established, and DNA probe-miRNA target hybrids and G4 were formed as they competed for available G-SNAP probes. Further, the G4 band appeared to resolve into two bands of similar intensities located close together. We attribute this to intermolecular G4 existing in two dominant configurations, presumably anti-parallel and parallel G4 intermolecular arrangements which exhibited subtle differences in electrophoretic mobilities. In the context of our study, we do not distinguish between these two bands but analyse them in tandem as the G4 band.

### Effect of K^+^ ion concentration on SNAP and G-SNAP discrimination of the *Cel-miR-54* core target against its isomiRs

G4s are known to be stabilized by K^+^ ions [35–37]. In this work K^+^ ions were chosen over Na+ ions as Na^+^ ions stabilize G-quadruplexes to a less extent than K^+^ ions and so we concluded that the decreased stability would not be beneficial to the detection properties of the G-SNAP probe [38]. Chen and co-workers' gold nanoparticles-mediated K^+^ detection platform showed a linear 1 μM to 1 mM K^+^ detection range [37], while Ambrus and co-workers, through the use of circular dichroism, demonstrated enhanced/larger characteristic G4 peaks from 0 mM to 100 mM K^+^, which suggested more extensive G4 formation [36]. In our study, we had observed that at 100 mM K^+^, the G4 band intensity was reduced when Cel-miR-54 was added (Fig 2B), thus prompting the study of G-SNAP hybridization with *Cel-54-miR* at higher K^+^ concentrations (300 mM and 500 mM in addition to 100 mM) and subjected to agarose gel electrophoresis after a 2-hour incubation. This also has the intended effect of investigating the enhancement of the gate-keeping property of the probes via more extensive G4 formation, which is the central premise of this work.

**Fig 3:**
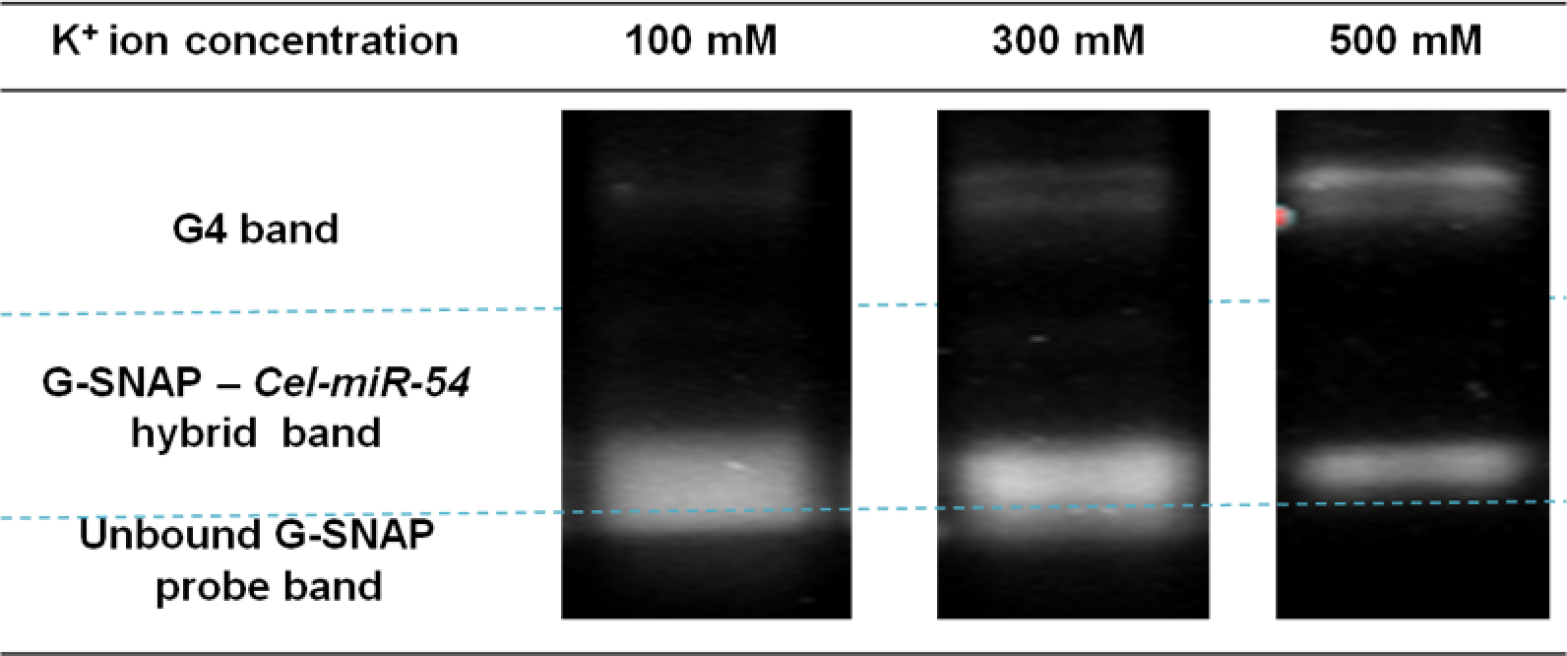
Representative gel images showing the change in the G-SNAP G4 band intensities at different K^+^ ion concentrations.

From Fig 3, we can make two principle observations: (1) As the K^+^ ion concentration was increased, the G4 band intensity was correspondingly increased. With more K^+^ ions present, they act as stabilizing ligands which enhance the formation of G4, as reflected through the stronger band intensities. A point of note is that this is not a straight-forward G4 formation as this process is complicated by the probe-miRNA hybridization, which necessitated higher K^+^ concentrations (>100 mM K^+^) for more extensive G4 formation. (2) As previously mentioned, the G4 forming equilibrium (*k*_*0*_) and G-SNAP-*Cel-miR-54* hybridization equilibrium (*k*_*1*_) are essentially in competition, and the increase in the intensity of the former was indeed accompanied by a decrease in the intensity of the latter. This is in spite of the fact that higher salt concentrations is known to favour more stable duplex hybridization, as higher cationic concentrations can better screen the negatively-charged DNA, bringing about more stable hybridization [39]. We conclude that this is evidence that G4 performs a gatekeeping role, in which the formation of G4 affected the DNA probe-miRNA target hybridizations. This in turn leads to more stringent discrimination against non-specific hybridization.

**Fig 4:**
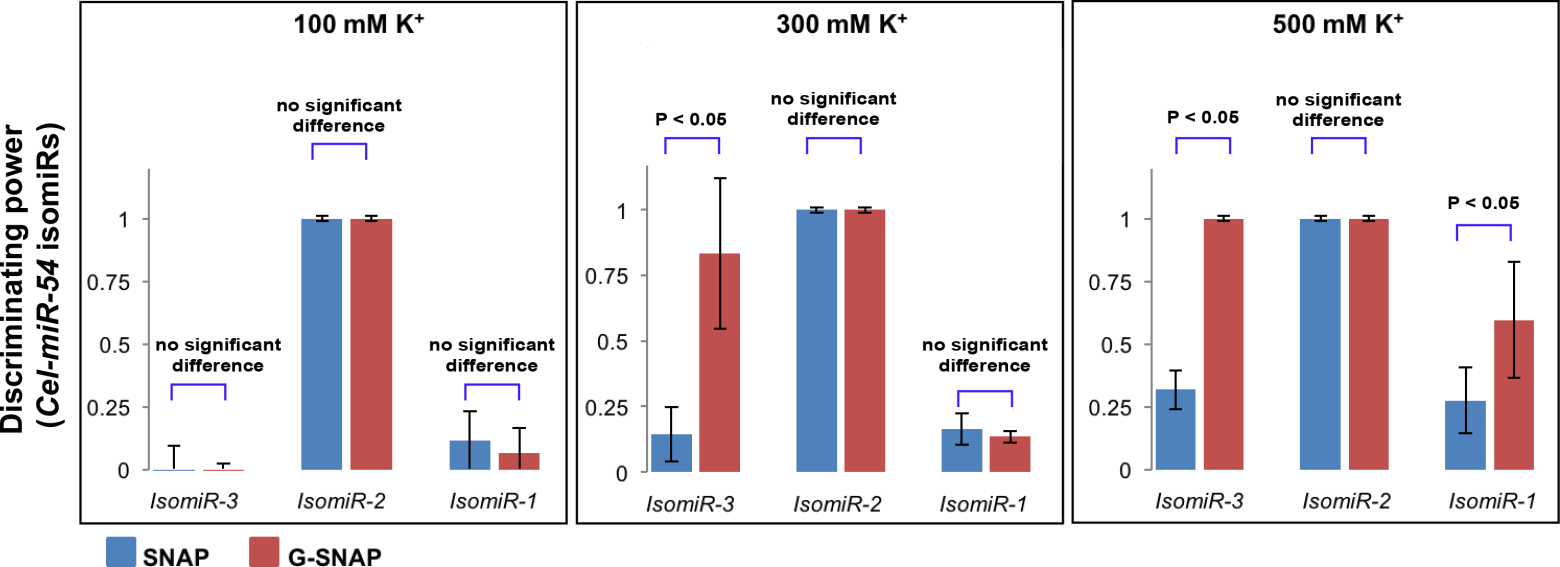
Summary of quantified hybrid band intensities (expressed as DP values) of *Cel-54* discriminated against mismatched isomiRs, by G-SNAP and SNAP, at 100 mM, 300 mM and 500 mM K^+^ ion, respectively. DP values were measured using Image-J software, from analysis of at least 3 independent repeats.

To establish the effect of G4 gatekeepers on discrimination of isomiRs, hybridization of SNAP and G-SNAP probes with *Cel-miR-54* core targets and its isomiRs were studied at different K^+^ ion concentrations (100 mM, 300 mM and 500 mM). Representative gel electropherograms showing the appearance of hybrids for the unbound probes for SNAP and G-SNAP against *Cel-miR-54* and *isomiRs-1*, *-2* and *-3* are represented in S1 Fig Each probe/target/K^+^ hybridization was repeated at least three times, and the intensities of the hybrid bands were quantified. S2 Fig shows the gel electropherogram of the SNAP hybridization with *Cel-miR-54* core target showing the significant degree of non-specific hybridizations that can exist in a direct probe-target hybridization scheme. By comparing the hybrid band intensity of the *Cel-miR-54* core target versus each isomiR target, DP ranging from 1.0 to 0.0 was determined with the results summarized in Fig 4.

At a K^+^ ion concentration of 100 mM, the results observed for both SNAP and G-SNAP were comparable (no significant statistical difference). Both SNAP and G-SNAP exhibited a more favourable discrimination against *IsomiR-2* (DP = 1.0) than *IsomiR-1* (DP = 0.10) *or IsomiR-3* (DP = 0.0). This is a result of the mismatch being in the middle of the recognition sequence for *IsomiR-2* which affords the least stable hybridization as the continuity of the base-pairing is disrupted. Now instead of a full13 bp base-pairing, as observed with the *Cel-miR-54* core target, there are two 6 bp overlaps. Both SNAP and G-SNAP could not discriminate between the *Cel-miR-54* core target and *IsomiR-3* (DP = 0.0), and there was also no significant difference in discrimination against *IsomiR-1*, with a DP of around 0.10 for both SNAP and G-SNAP. This could be attributed to the mismatch being located near the ends of the recognition sequence such that the specific base-pairing (12 bp) had already rendered stable hybrid formation. This thus mitigated stringent discrimination against these mismatched isomiRs.

At a K^+^ ion concentration of 300 mM, the DP against *IsomiR-2* remained at 1.0 for both SNAP and G-SNAP probes. *IsomiR-1* discrimination (DP = 0.15) was also comparable for both SNAP and G-SNAP probes. However, G-SNAP probe did show a significantly enhanced discrimination against *IsomiR-3* (DP = 0.80) compared to that of the SNAP probe (DP = 0.15), with a p-value < 0.05. In *IsomiR-3*, the mismatch is located towards the 5' end of the miRNA (Fig 1). At higher (300 mM) K^+^, the destabilization due to the sequence mismatch at the miRNA 5' end, coupled by the enhancement of the G4-forming PG-tract (at higher K^+^ concentration) towards the 5' end of the G-SNAP probe (corresponding to the miRNA 3' end) could combine to bring about more stringent discrimination.

At a K^+^ ion concentration of 500 mM, the SNAP probe was still able to discriminate against *IsomiR-2* with high fidelity (DP = 1.0) while for *IsomiR-3* and *IsomiR-1* DP was 0.30 and 0.25, respectively. Conversely, the G-SNAP probe showed high fidelity discrimination against both *IsomiR-3* and *IsomiR-2* (DP = 1.0), while that for *IsomiR-1* improved to 0.60. The p-values calculated for the DP of G-SNAP against SNAP for *IsomiR-3* and *IsomiR-1* were both less than 0.05. Even though the mismatch in *IsomiR-1* is located at the 3' end of the miRNA, the increase in K^+^ ion concentration enhanced the G4 formation (evidenced in Fig 3). This competitive G4-forming equilibrium (*k*_*0*_, Fig 1) can lead to further reduction in DNA probe-miRNA target hybridizations, especially the non-specific hybridizations of mismatched IsomiRs (*k*_*2*_, *k*_*3*_ and *k*_*4*_, Fig 1) Overall, the increase in K^+^ ion concentration corresponded to improved discrimination against mismatched targets, which was particularly pronounced for G-SNAP.

The effect of G4 on the hybridization of G-SNAP and miRNA target and discrimination against the IsomiR must be distinguished from the situation wherein lower amounts of probes were purposely added, or optimized, to reduce non-specific hybridizations. In this case, the probes and *Cel-miR-54* core targets were in equimolar amounts. It is because of the inherent ability of the G-SNAP probes to assemble into G4 and the establishment of a competing G4 equilibrium that hybridization was affected. This is especially true at higher K^+^ ion concentrations where non-specific hybridizations are most affected, as these mismatched hybrids are not as stable as hybrids formed from *Cel-miR-54*. This therefore results in a less favoured equilibrium and forms the basis for the stringency of the detection and discrimination.

### Translocation of the position of the G4 gate-keeper and its effect on isomiR discrimination

As previously mentioned, the relative position of the PG tract from the recognition sequence is a design parameter which affects the G4 disruption of DNA-miRNA base-pairing [33]. To observe the importance of the position of the PG tract within the G-SNAP probe the PG tract was translocated by one base pair towards the 3’ end (G-SNAP-Alt probe (Table 1)). This design not only allowed for the formation of G4 at a different location along the probe sequence, but also ensured that one of the guanine bases in the PG tract was part of the miRNA recognition sequence.

**Fig 5:**
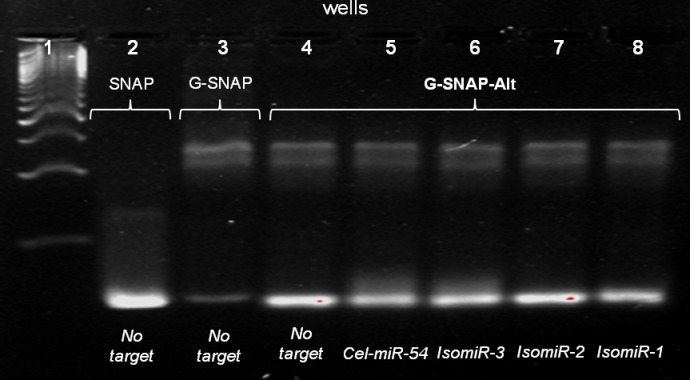
SYBR-safe pre-stained agarose gel image showing the hybridization of G-SNAP-Alt against *Cel-miR-54* core and its mismatched isomiRs, at 500 mM K^+^.

Fig 5 depicts the SYBR-safe pre-stained agarose gel electropherogram showing the bands for the hybridizations of the G-SNAP-Alt probe against the *Cel-miR-54* core target and the *IsomiR-3*, *IsomiR-2*, *IsomiR-1* targets. Similar to G-SNAP (Fig 5, Lane 3) without the target the G-SNAP-Alt probe showed both unbound probe and G4 bands (Fig 5, Lane 5). However, the unbound probe band for G-SNAP-Alt showed stronger intensity than that for G-SNAP, while the G4 band intensity was slightly reduced in intensity. This suggested that the propensity for G4 formation was dependent on where the PG tract was located. However, the G4 band's presence indicated that its gate-keeping potential remained intact. From Fig 5 (Lanes 4 to 8), G-SNAP-Alt formed probe-target hybrid bands with *Cel-miR-54* target (Fig 5, Lane 5) and *IsomiR-3* (Fig 5, Lane 6), while for *IsomiR-2* and *IsomiR-1* (Fig 5, Lanes 7 and 8, respectively), only unbound probe and G4 bands were observed. These highlight the importance of the position of the PG tract. Unlike G-SNAP, the one base overlap with the target sequence in G-SNAP-Alt was observed to dramatically affect the hybridization outcome for both perfectly-matched (*Cel-miR-54*) and mismatched (isomiR) targets as not only were non-specific hybridizations not observed (Fig 5, Lanes 6, 7 and 8), the perfectly-matched *Cel-miR-54* also did not appreciably hybridize with G-SNAP-Alt (Fig 5, Lane 5). Here, the overlap of the PG-tract with the target recognition sequence and the formation of G4 may more directly, and extensively, negate hybridization.

### Enzymatic extension by M-MLV RT

**Fig 6:**
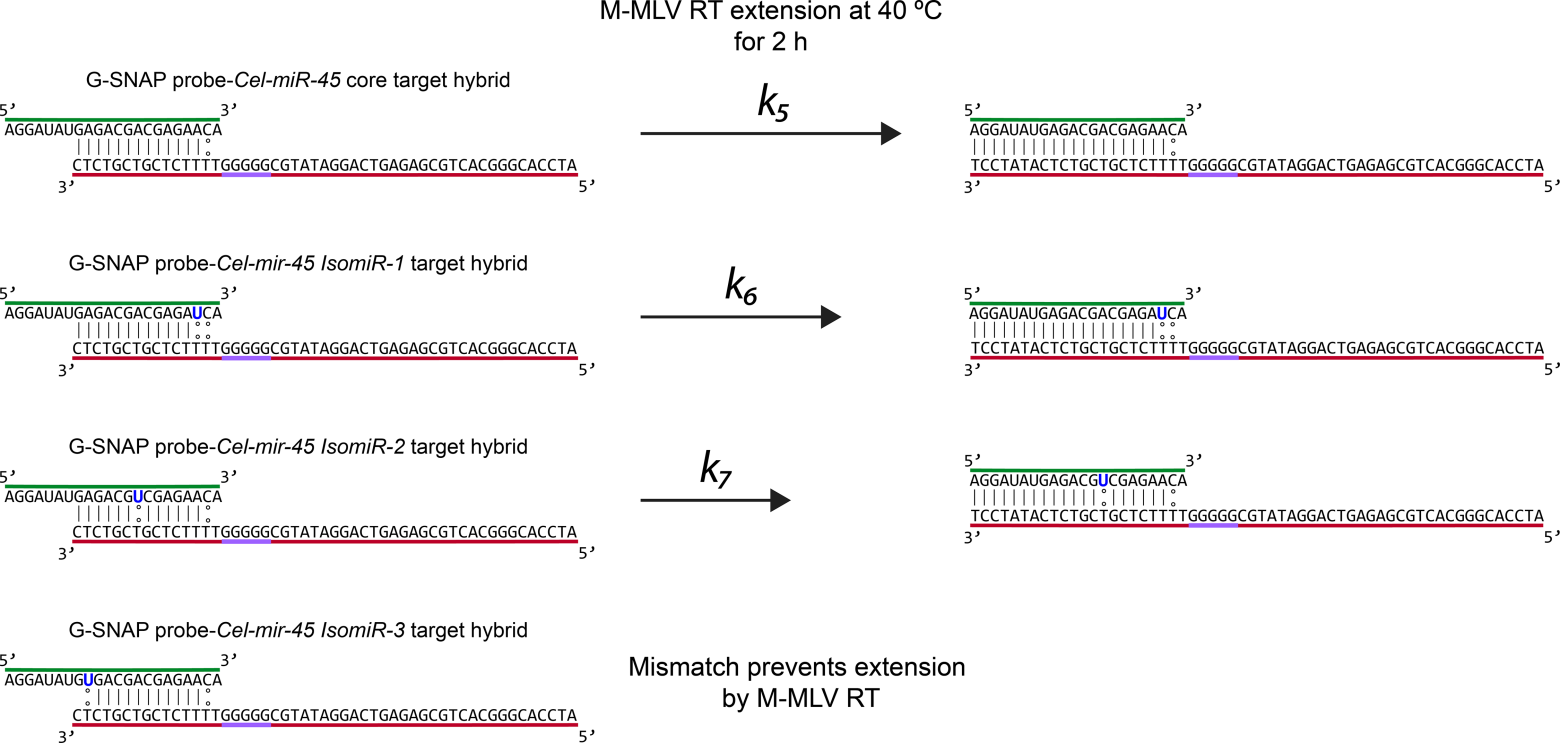
Working principle of how M-MLV RT extends G-SNAP probes hybridised with *Cel-miR-54*, *IsomiR-1*, *IsomiR-2*, and *IsomiR-3*. *IsomiR-3* however does not allow for extension due to the presence of the mismatch towards the 5' end of the miRNA (correspondingly at the 3’ end of the G-SNAP probe).

M-MLV RT, a DNA/RNA reverse transcriptase, was introduced to extend the 3' end of the hybridized DNA probes to increase DNA probe-miRNA target base-pairing. The extension cannot occur in the opposite direction (5' to 3') as the mismatch is at the 21^st^ nucleotide (Fig 6).

The addition of an enzymatic extension step also allowed for amplification of signal and more sensitive detection. Such strategy is utilized in many detection platforms [40], and it is important to determine if the G4-modified probes can maintain their gate-keeping property even after enzymatic amplification. In addition, by extending the DNA probe length from the 3' to 5' in order to match the over-hang of the target miRNA, and by identifying the degree of electropherogram band shifting and change in intensity, it is possible to determine approximately where the mismatch might be present. It was postulated that mismatches that appear closer the 5' of the target (miRNA) are less likely extended, therefore there should be no appearance of a higher molecular weight band or decreased intensity of the band will be observed.

The closer the mismatch is to the 3' (or at least far enough from the nucleation site of the M-MLV RT with the DNA probe-miRNA target hybrid) the more likely it is for the appearance of the higher molecular weight band and increased intensity as the hybrid becomes more stable, i.e., the more stable the hybrid is the more likely for extension to occur. G-SNAP and G-SNAP-Alt probes were hybridized with the *Cel-miR-54* core target and the mismatched isomiR targets, followed by M-MLV RT extension.

**Fig 7:**
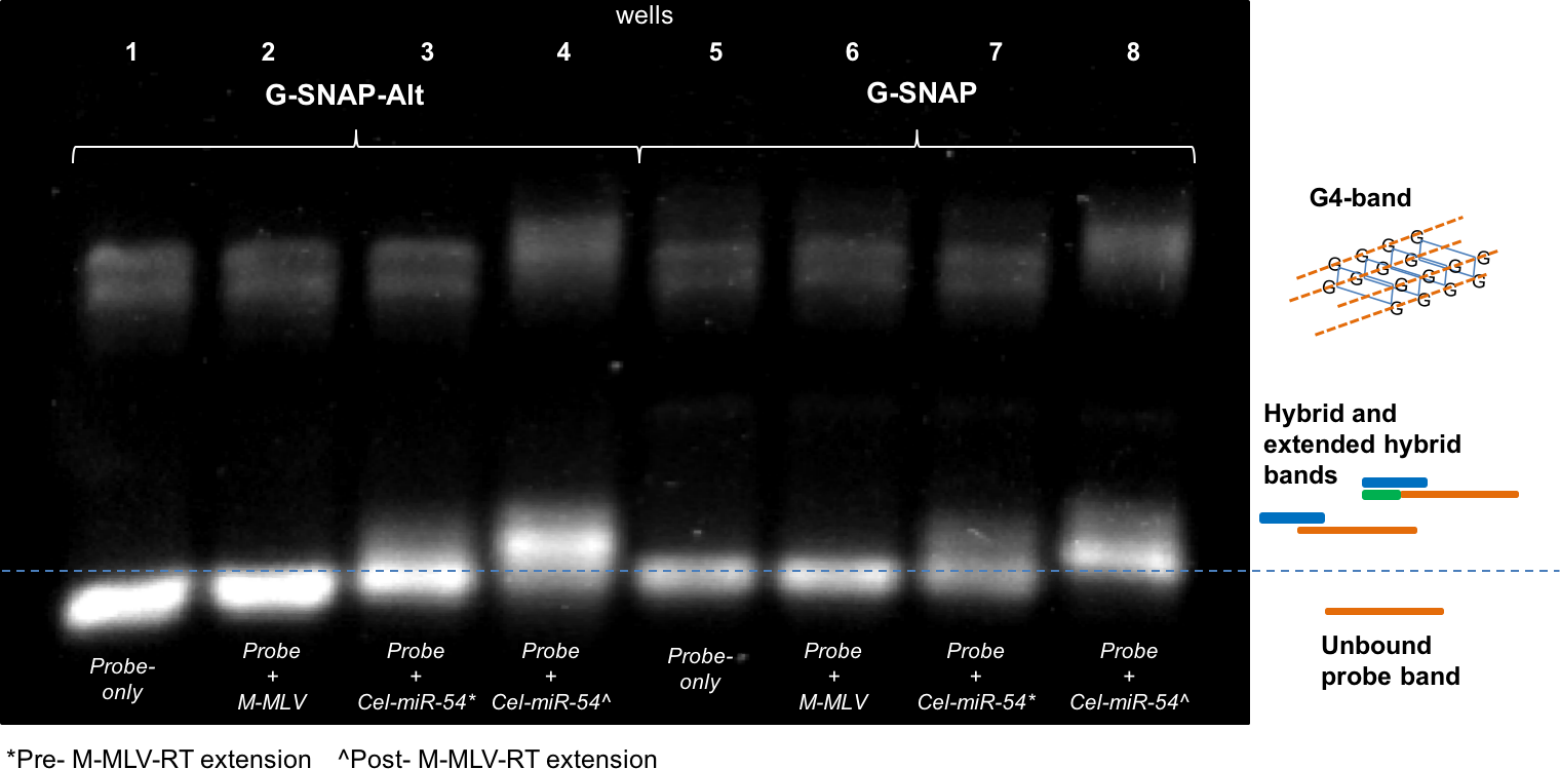
SYBR-safe pre-stained agarose gel image showing the effect of M-MLV RT extension on G-SNAP and G-SNAP-Alt probes (probes-only, and probes hybridized with *Cel-miR-54*).

From the gel electropherograms (Fig 7) two bands were observed. The probe-only controls for G-SNAP-Alt (Fig 7, Lane 1) and G-SNAP (Fig 7, Lane 5) showed no appreciable change in intensity upon M-MLV RT extension (Fig 7, Lanes 2 and 6, respectively). However, the intensities of the DNA probe-*Cel-miR-54* target hybrid bands of both the G-SNAP-Alt (Fig 7, Lanes 3 and 4) and G-SNAP (Fig 7, Lanes 7 and 8) were increased and became more defined. This enhanced intensity implies that the enzymatic extension was most pronounced for the DNA probe-miRNA target hybrids. as the increased base-pairing will increase the intensity of the bands as more SYBR-safe molecules intercalate. The band intensity can also be used to ascertain whether or not extension was successful–allowing for potential assignment of mismatch location in the *IsomiRs*, as discussed further.

From the gel electropherograms (Fig 7) two bands were observed. The probe-only controls for G-SNAP-Alt (Fig 7, Lane 1) and G-SNAP (Fig 7, Lane 5) showed no appreciable change in intensity upon M-MLV RT extension (Fig 7, Lanes 2 and 6, respectively). However, the intensities of the DNA probe-*Cel-miR-54* target hybrid bands of both the G-SNAP-Alt (Fig 7, Lanes 3 and 4) and G-SNAP (Fig 7, Lanes 7 and 8) were increased and became more defined. This enhanced intensity implies that the enzymatic extension was most pronounced for the DNA probe-miRNA target hybrids. The observed band retardation approximately corresponded to the expected 7 bp increase in molecular weight. as more SYBR-safe molecules intercalate. The band intensity can also be used to ascertain whether or not extension was successful–allowing for potential assignment of mismatch location in the *IsomiRs*, as discussed further.

The higher band in the DNA probe-*Cel-miR-54* target hybrid after upon M-MLV RT extension of both the G-SNAP-Alt (Fig 7, Lanes 3) and G-SNAP (Fig 7, Lanes 8) is associated with G4. This G4 band now appears to have an increased weight relative to the non-extended DNA probe-miRNA hybrid. This increased weight may be due to the equilibrium shift that would occur at 40°C (temperature used for extension). It is expected that after extension occurs the hybrids melts back into single stranded nucleic acids and may either hybridize, or the probes could be incorporated, into a G4 structure.

**Fig 8:**
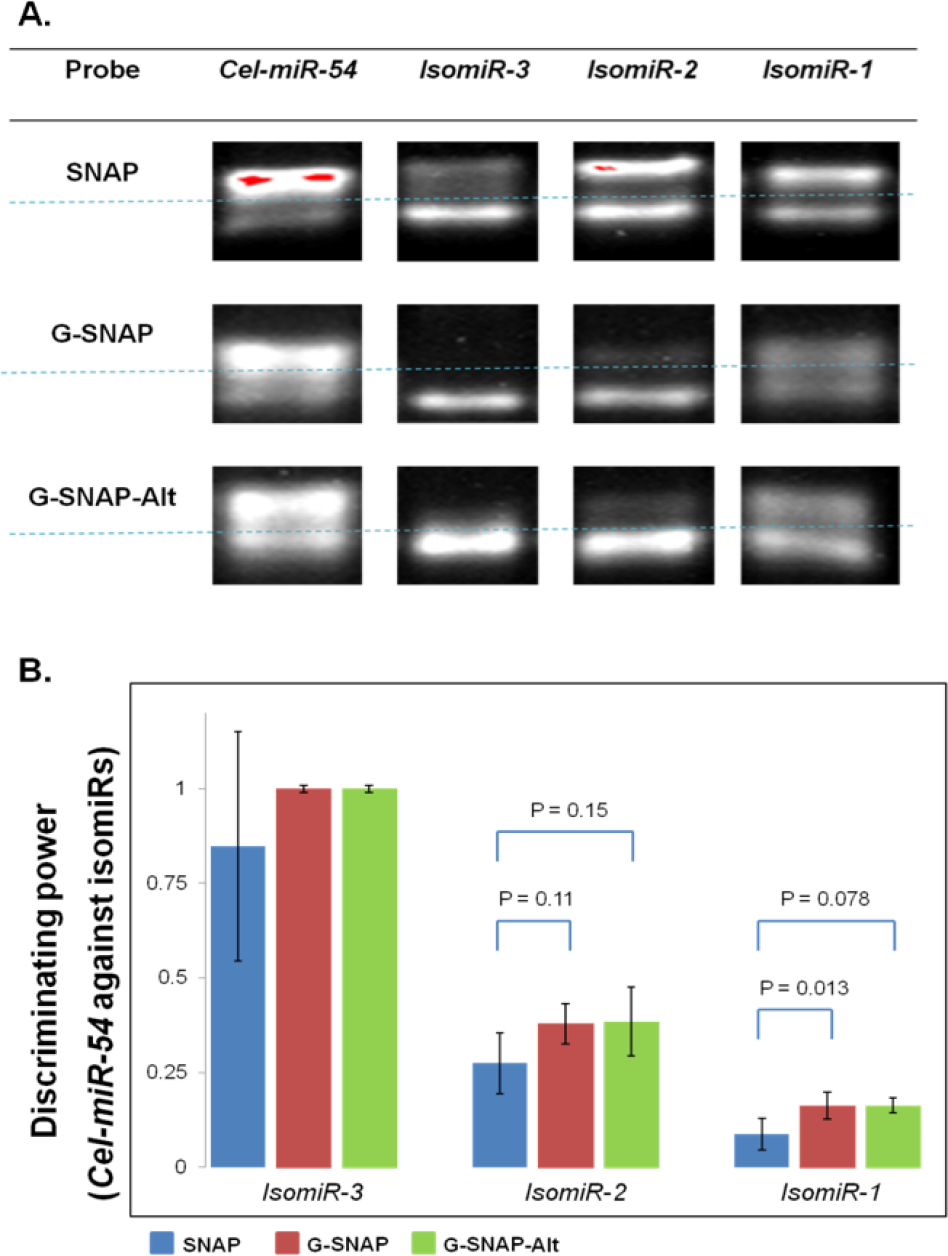
(A). SYBR-safe pre-stained agarose gel image of unbound probe and hybrid bands after *Cel-miR-54* and its isomiRs hybridization with SNAP, G-SNAP and G-SNAP-Alt, followed by and M-MLV RT extension; (B). DP of SNAP, G-SNAP and G-SNAP-Alt against mismatched isomiRs, after M-MLV RT extension.

We then expanded the study to the mismatched isomiRs, and M-MLV extension was again found to increase the intensities of hybrid bands across SNAP, G-SNAP and G-SNAP-Alt probes, as exhibited by the gel images shown in Fig 8A.

However, an increase in the hybrid band intensities was observed not just for both *Cel-miR-54* core target but also for mismatched isomiRs. There was no extension for *IsomiR-3* (DP = 1.0, Fig 7B*)*, which could be explained by the proximity of the mismatch being very close to the 3' end of the DNA in the DNA probe-miRNA target hybrid (Table 1, Fig 1) and preventing the M-MLV RT from extending the DNA probe. Previously, all three probes showed a DP of 1.0 for *IsomiR-2* (Fig 4) due to the mismatch occurring in the center of the DNA probe-miRNA target hybrid and destabilizing the hybrid considerably more than the perfect match. However, after M-MLV RT extension, a relatively intense *IsomiR-2* hybrid band was observed for SNAP, while faint hybrid bands were also observed for G-SNAP and G-SNAP-Alt (Fig 8A). This is due to the stabilization that comes from the increased length of the probes. As the probe is extended the mismatch no longer has a significant impact on the T_m_ of the whole strand. This is what would be expected when the mismatch is far enough along the hybrid to not interfere with the M-MLV RT but still forms an unstable hybrid. The calculated p-values are relatively weak, at 0.11 and 0.15 for SNAP and G-SNAP, and SNAP and G-SNAP-Alt, respectively. Finally, *IsomiR-1* appeared to be the hardest to discriminate as the calculated DP was low, at 0.08 for SNAP and 0.16 for both G-SNAP and G-SNAP-Alt (Fig 8B). This follows on from the previous two mismatch extensions (*IsomiR-2 and IsomiR-3*), in that the mismatch does not interfere with the M-MLV RT and does not destabilize the DNA probe-miRNA target hybrid and therefore the rate will be *k*_*6*_ > *k*_*7*_. When calculated for significance, both G-SNAP and G-SNAP-Alt were found to be significantly different (p = 0.013 and 0.078 respectively) when compared to SNAP. Despite the action of M-MLV RT decreasing the DP, probes modified with G4-forming sequence still performed better than SNAP, which argues for the incorporation of this gate-keeping ability in the probe design.

Another significance here is that it is possible to approximately determine where the mismatch is located in this particular DNA probe-miRNA target hybrid system. The data indicates that if the mismatch is close to the 3' region that there will be no extension and no bands formed (e.g., *IsomiR-3*); if the mismatch is located near the center then a low extension rate will be observed and low intensity bands will be observed; and if the mismatch is closer the 5' region of the strand to be extended than the stability of the hybridization will allow for a band of moderate intensity to be observed.

### Conclusion

Our results showed that incorporating PG tracts with G4-forming ability into the design of molecular probes for miRNA targets addressed the problem of non-specific hybridizations typically associated with isomiRs, and also achieved more stringent discrimination of miRNA core target (in this case, *Cel-miR-54*) over mismatched isomiRs. We also showed that the location of the PG tract in the probes is another important design consideration as the target detection and readout clarity are both affected. Finally, the reverse transcriptase-mediated extension of probe-target hybrids also allowed the different isomiRs to be discriminated from one another based on the locations of the mismatch.

## Supporting information

S1 Fig Representative gel images showing the unbound probe and hybrid probe bands of SNAP and G-SNAP, when hybridized with *Cel-miR-54* and its isomiRs, at different K^+^ ion concentrations. (PDF)

S2 Fig SYBR-safe pre-stained agarose gel images showing hybridization between SNAP and perfect-match (*Cel-miR-54*) and mismatched isomiRs. (PDF)
