# Supplementary material for: Hi-fidelity discrimination of isomiRs using G-quadruplex gatekeepers
Source: PLoS One. 2017 Nov 16;12(11):e0188163. doi: 10.1371/journal.pone.0188163 (PMC5690596; doi:10.1371/journal.pone.0188163)
Supplement: S1 Fig — (PDF) [file pone.0188163.s001.pdf]

	**SNAP**				**G-SNAP**			
K^+ ion concentration	*Cel-miR-54*	*IsomiR-3*	*IsomiR-2*	*IsomiR-1*	*Cel-miR-54*	*IsomiR-3*	*IsomiR-2*	*IsomiR-1*
100 mM	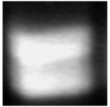	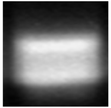	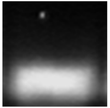	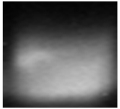	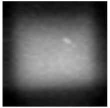	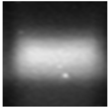	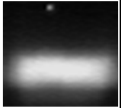	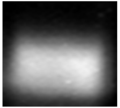
300 mM	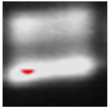	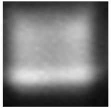	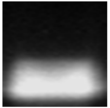	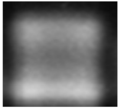	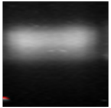	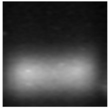	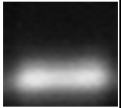	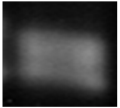
500 mM	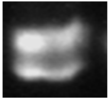	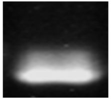	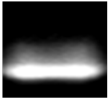	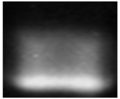	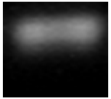	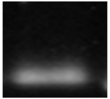	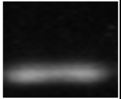	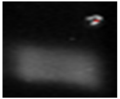
