# Supplementary figures and images for: Hi-fidelity discrimination of isomiRs using G-quadruplex gatekeepers
Source: PLoS One. 2017 Nov 16;12(11):e0188163. doi: 10.1371/journal.pone.0188163 (PMC5690596; doi:10.1371/journal.pone.0188163)

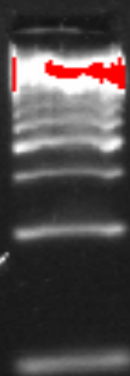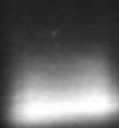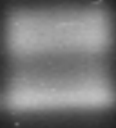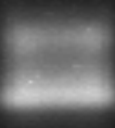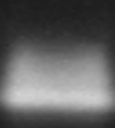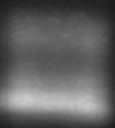

Supplement: S2 Fig — (PDF) [file pone.0188163.s002.pdf]
